# STW 5 Herbal Preparation Modulates Wnt3a and Claudin 1 Gene Expression in Zebrafish IBS-like Model

**DOI:** 10.3390/ph14121234

**Published:** 2021-11-28

**Authors:** Monica Piccione, Nicola Facchinello, Sandra Schrenk, Marco Gasparella, Surajit Pathak, Ramy M. Ammar, Sabine Rabini, Luisa Dalla Valle, Rosa Di Liddo

**Affiliations:** 1 Department of Pharmaceutical and Pharmacological Sciences, University of Padova, 35131 Padova, Italy; monica.piccione@phd.unipd.it (M.P.); sandra89.schrenk@gmail.com (S.S.); 2 Department of Biology, University of Padova, 35131 Padova, Italy; nicola.facchinello@unipd.it (N.F.); luisa.dallavalle@unipd.it (L.D.V.); 3 Department of Pediatric Surgery, Ca’ Foncello Hospital, 31100 Treviso, Italy; marco.gasparella@unipd.it; 4 Department of Medical Biotechnology, Faculty of Allied Health Sciences, Chettinad Hospital and Research Institute (CHRI), Chettinad Academy of Research and Education (CARE), Kelambakkam, Chennai 603103, Tamil Nadu, India; surajit.pathak@gmail.com; 5 BAYER Consumer Health, Global Medical Affairs, 64295 Darmstadt, Germany; ramy.ammar@bayer.com (R.M.A.); sabine.rabini@bayer.com (S.R.); 6 Department of Pharmacology and Toxicology, Faculty of Pharmacy, Kafrelsheikh University, Kafr-El Sheikh 33516, Egypt

**Keywords:** IBS, STW 5, NF-kβ, Wnt signaling, claudin 1, wnt3a, zebrafish

## Abstract

Aim: Irritable bowel syndrome (IBS) is a functional bowel disorder characterized by chronic abdominal pain and stool irregularities. STW 5 has proven clinical efficacy in functional gastrointestinal disorders, including IBS, targeting pathways that suppress inflammation and protect the mucosa. Wnt signaling is known to modulate NF-kβ-dependent inflammatory cytokine production. This sparked the idea of evaluating the impact of STW 5 on the expression of inflammatory-response and Wnt/β catenin-target genes in an IBS-like model. Main methods: We used zebrafish and dextran sodium sulfate (DSS) treatment to model IBS-like conditions in vivo and in vitro and examined the effects of subsequent STW 5 treatment on the intestines of DSS-treated fish and primary cultured intestinal and neuronal cells. Gross gut anatomy, histology, and the expression of Wnt-signaling and cytokine genes were analyzed in treated animals and/or cells, and in controls. Key findings: DSS treatment up-regulated the expression of *interleukin-8*, *tumor necrosis factor-α*, *wnt3a*, and *claudin-1* in explanted zebrafish gut. Subsequent STW 5 treatment abolished both the macroscopic signs of gut inflammation, DSS-induced mucosecretory phenotype, and normalized the DSS-induced upregulated expression of il10 and Wnt signaling genes, such as *wnt3a* and *cldn1* in explanted zebrafish gut. Under inflammatory conditions, STW 5 downregulated the expression of the pro-inflammatory cytokine genes *il1β*, *il6*, *il8*, and *tnfα* while it upregulated the expression of the anti-inflammatory genes *il10* and *wnt3a* in enteric neuronal cells in vitro. Significance: Wnt signaling could be a novel target for the anti-inflammatory and intestinal permeability-restoring effects of STW 5, possibly explaining its clinical efficacy in IBS.

## 1. Introduction

Irritable bowel syndrome (IBS) is one of the most common functional bowel disorders and is defined by chronic or recurrent abdominal pain, stool irregularities, and bloating [1,2,3] in the absence of known organic pathology.

IBS-related morpho-functional changes have been reported in both the intestinal epithelial barrier (e.g., goblet cell hyperplasia, increased paracellular permeability, lower expression of tight junctions, altered expression of claudins) and the enteric neuromuscular compartment (e.g., decreased muscle layer thickness, reduced entero-endocrine cell activity, altered circulating levels of serotonin (5-HT3)).

Currently, the management of IBS patients is complex and the available treatments for this functional disorder include pharmacological approaches combined with a healthy lifestyle. Among the treatment options, there are antispasmodics, antidepressants, opioid receptor agonists, 5-HT3 receptor antagonists, anti-inflammatory agents, antibiotics, probiotics, laxatives, and prosecretory agents [4,5]. Besides providing no definitive solution for IBS, these drugs cause remarkable adverse effects and/or show low efficacy in controlling multiple symptoms simultaneously [6,7,8,9,10,11]. As alternative options to chemically defined medications, herbal medicinal products have demonstrated efficacy in the treatment of functional bowel disorders [12]. Containing a variety of active components, they offer the advantage of targeting various underlying pathways but, similarly to traditional drugs, they may also cause adverse effects [13]. Thus, appropriate toxicological studies and pharmacovigilance programs are required to ensure that patients are not exposed to unjustifiable risks. Belonging to this class of medicines, STW 5 is a hydro-ethanolic liquid preparation composed of the nine medicinal herbs *Iberis amara*, *Angelicae radix*, *Cardui mariae fructus*, *Chelidonii herba*, *Liquiritiae radix*, *Matricariae flos*, *Melissae folium*, *Carvi fructus*, and *Menthae piperitae folium*. Several prospective, controlled clinical trials have confirmed the clinical efficacy of STW 5 in managing various functional gastrointestinal disorders, including functional dyspepsia and IBS [14,15,16,17,18,19]. Through several mechanisms of action [20,21], STW 5 has demonstrated the ability to induce (i) spasmolytic/tonicizing effects on intestinal smooth muscle, (ii) anti-inflammatory effects, (iii) pain reduction by modulating GI sensory afferent neuronal signaling, and (iv) regulation of the microbiome [16,22,23,24].

Growing evidence suggests that dysregulated activation of Wnt signaling in the enteric nervous system [25], stromal compartment [9,26], immune cells [27], and epithelium [28] is involved in various aspects of the pathogenesis of functional GI disorders, including visceral hypersensitivity, altered gut motility and low-grade gut inflammation [29]. Intersecting with major inflammatory pathways, the Wnt pathway has both anti- and pro-inflammatory effects in the gut. In particular, canonical Wnt downregulates NF-kβ-dependent transcription of the interleukins (*IL*)*-1β*, *IL-6*, *IL-8*, and tumor necrosis factor (*TNF*)*-α* [30,31]. Moreover, as reported by Manicassamy et al. [32], the activation of β-Catenin in dendritic cells contributes to the switch from immunity to tolerance, stimulating the expression of the anti-inflammatory NF-kβ target gene IL-10. As previously reported by our group, the Wnt receptor Frizzled-9 and its ligand Wnt3a are expressed in the rat myenteric plexus of the enteric nervous system (ENS) [25], and the alteration of adherent junctions in ENS cells in vitro induces an aberrant accumulation of β-Catenin [33]. In contrast, the activation of pro-inflammatory mediators seems to be dependent on the non-canonical Wnt pathway [34].

Considering that Wnt plays a role in the modulation of inflammatory cytokine production, such as through the NF-kβ signaling pathway [35], it is conceivable that STW 5 alleviates IBS by modulating the expression of inflammatory-response and Wnt-target genes. Testing this hypothesis for the first time, in this study we assessed in vivo and in vitro zebrafish models of IBS-like conditions induced by treatment with 0.5% dextran sodium sulfate (DSS) for 8 h. Sharing numerous gut functions and immune genes with mammals, the zebrafish has been proposed as a model of intestinal inflammation and injury [36]. Based on the evidence acquired in our laboratory and previously reported [37], the inflammation induced by DSS for 8 h resembles the gastrointestinal alterations observed in IBS patients, such as (i) low-grade gut inflammation; (ii) goblet cell dysplasia; and (iii) upregulated transcription of both *claudin1*, as is typically observed in IBS-C patients [38,39], and *wnt3a*, as is commonly observed during gut inflammation [40].

## 2. Results

### 2.1. In Vivo Model of IBS and Impact of STW 5 on Morphological Changes

**Figure 1:**
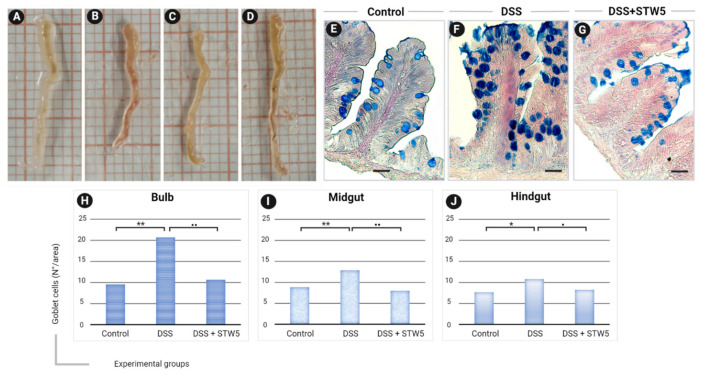
Macroscopic features of gut explants from zebrafish under (**A**) resting conditions, (**B**) after 8 h of treatment with 0.5% dextran sodium sulfate (DSS), (**C**) after 16 h of treatment with 0.12 mg/Ml STW 5, and (**D**) after DSS + STW 5 treatment. (**E**–**G**) Tissue sections of zebrafish gut (bulb) stained with Alcian blue showing the distribution and density of goblet cells (blue) under resting conditions, DSS- or DSS+STW5-treatment. (**H**–**J**) Relative number of goblet cells in bulb, midgut, and hindgut explants. The quantification was performed using five different slides of each part of the intestine from three animals (*n* = 15 sections total for bulb, midgut, and hindgut). Data are expressed as the mean number of goblet cells ± standard deviation (SD). Statistical significance was calculated using the Wilcoxon test comparing DSS-treated or DSS+STW 5–treated samples with controls. * *p* ≤ 0.05; ** *p* ≤ 0.01. DSS+STW 5–treated samples vs. DSS (^•^ *p* ≤ 0.05; ^••^ *p* ≤ 0.01).

**Figure 2:**
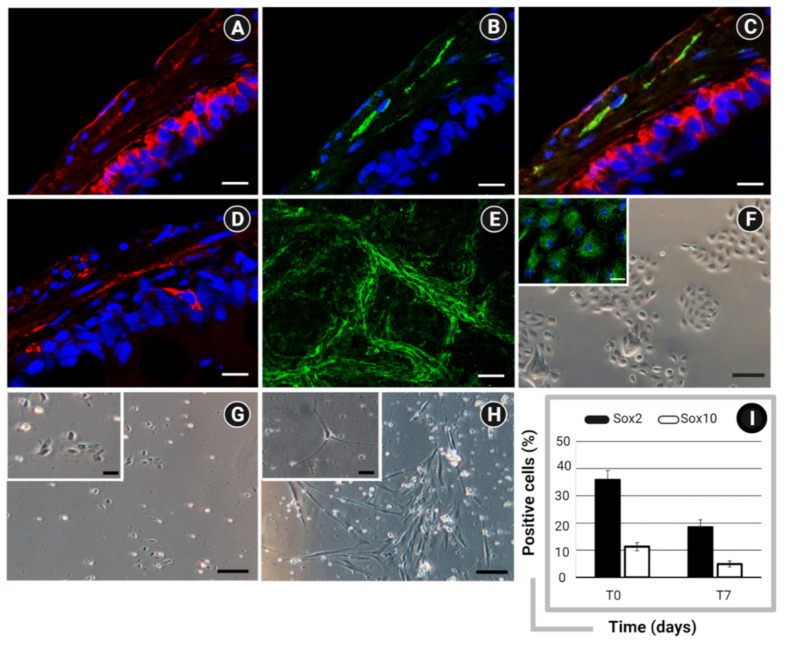
Immunofluorescent staining showing the expression of (**A**) Frizzled 9 (red) within the epithelial and neuromuscular compartments; (**B**) PAN neuronal marker (green) within the neuromuscular layer; and (**C**) both markers (Frizzled 9 and PAN neuronal marker) in colocalization (yellow); and (**D**) Wnt3a within the epithelium and neuromuscular layer. Nuclear counterstaining was performed using DAPI (blue). (**E**) Whole-mount immunofluorescent staining of zebrafish ENS network by detection of PAN neuronal marker (green) Bar: 50 µm. Optical microscopy of (**F**) primary intestinal epithelial cells, characterized by the expression of Pan-cytokeratin (left corner, green); ENS cultures at (**G**) 24 h and (**H**) 7 days from isolation. Bar: 100 µm (200 µm in left corner). (**I**) Flow cytometry analysis of Sox2 and Sox10 in ENS cells at the time of isolation (T0) and after culturing for 7 days (T7). Data are expressed as percentage (%) of positive cells ± standard deviation (SD). Created with BioRender.com, accessed on 31 October 2021.

The macroscopic evaluation of selected intestine explants is presented in Figure 1A–D. Compared with their controls, DSS-treated zebrafish showed increased blood flow and accumulation of fluids (Figure 1A,B). In contrast, no signs of visceral inflammation were visible in samples isolated from zebrafish treated with STW 5 alone (Figure 1C) or in combination with DSS (Figure 1D). As an increased number of mucus-producing cells is commonly considered to indicate active inflammation, we counted the goblet cells in bulb, midgut, and hindgut explants. Goblet cells are mucus-secreting epithelial cells that act as the first line of defense against dangerous physical and chemical factors from ingested food, microbes, and microbial products [41,42]. As previously reported by Cheng et al. [43], small intestine and colon samples of patients with constipation- or diarrhea-predominant IBS exhibit changes in goblet cells and altered mucus secretion. In our study, STW 5 treatment reversed the inflammatory response by reducing the numbers of goblet cells in the bulb, midgut, and hindgut to the levels detected in control samples (Figure 1E–J). The overall organization of the enteric nervous system, connective tissue, and smooth muscle layers in zebrafish intestine is comparable to that of mammals, though the former has a less complex lamina propria and lacks a submucosa [44]. Here, immunofluorescence revealed Frizzled-9 in both the epithelium and the muscle layer (Figure 2A) of the zebrafish gut. Interestingly, Frizzled-9 co-localized with the PAN-neuronal marker (Figure 2B,C), suggesting it is expressed in myenteric neurons. As shown in Figure 2D, Wnt3a was distributed within the epithelial and muscle layers, providing evidence that epithelial cells (Epc) and ENS cells (Figure 2E–H) are responsive to canonical Wnt signaling.

### 2.2. In Vitro Models of IBS

In vitro models of IBS-like conditions were assessed, and culture conditions were defined to preserve cellular functionality. EP cells exhibited a typical organization in colonies, polygonal shape, and cytokeratin expression (Figure 2F). Freshly isolated ENSc included both immature (Figure 2G) and mature (Figure 2H) cells. Flow cytometry revealed specific immunoreactivity for Sox2, a known marker of neuronal progenitors [45,46], and Sox10, a marker of neural crest cells [47] (Figure 2I).

### 2.3. In Vivo Response to STW 5

**Figure 3:**
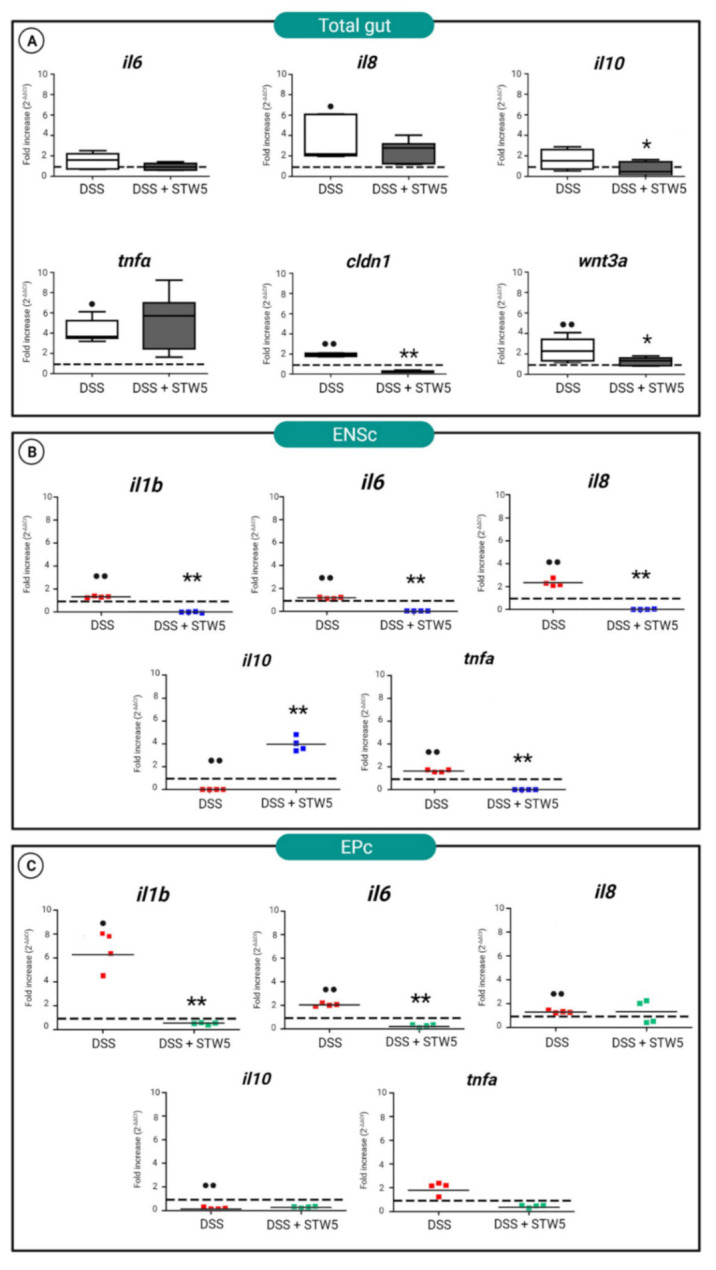
(**A**) Quantitative PCR analysis of *il6*, *il8*, *il10*, *tnfα*, *wnt3a*, and *cldn1* genes in explanted zebrafish total guts. The tissues were explanted from animals treated for 8 h with DSS (controls) or incubated with STW 5 for 16 h after DSS stimulation. qRT-PCR of *il6*, *il8*, *il10*, and *tnfα* in (**B**) ENS cultures (ENSc), and (**C**) epithelial cells (EPc). Expression levels were determined as relative to the expression of the housekeeping gene *ef1α*, normalized to controls, and calculated using the ΔΔCt method based on the equation 2^–ΔΔCt^ ± standard deviation. In the graphs, the expression level of controls was set to 1 and indicated with a dotted line. Statistical significance was calculated using the Wilcoxon test. Samples vs. controls (^•^ *p* ≤ 0.05; ^••^ *p* ≤ 0.01). Samples vs. DSS (* *p* ≤ 0.05; ** *p* ≤ 0.01).

Several studies have investigated mucosal levels of cytokines in IBS patients. *In vitro* and in vivo experiments have demonstrated that the secretion of IL-10, an anti-inflammatory cytokine, is depressed at baseline, while IL-1β, IL-6, IL-8, IL-12, and TNF-α are elevated. The treatment of zebrafish with 0.5% DSS for 8 h (Figure 3A) induced an inflammatory state characterized by increased *il8*, *tnfa*, *cldn1*, and *wnt3A* mRNA levels. No significant changes were observed in the mRNA levels of *il6* or *il10*. The inflammatory response is critical for fighting insults such as pathogen invasion or tissue damage, but if inflammation becomes chronic it is often detrimental to the host. Accordingly, the balance between protective and degenerative inflammation is gaining attention from a therapeutic point of view [48]. Several mechanisms operate to ward off exaggerated neuroimmune responses or to appropriately enhance a protective immune response [49]. One of these mechanisms is the production of anti-inflammatory cytokines, such as IL-10. Under the IBS-like conditions induced by DSS, *il10* and *Wnt* signaling genes, such as *wnt3a* and *cldn1*, were negatively regulated by STW 5 treatment. This observation highlights the ability of STW 5 to favor the resolution of inflammatory processes that interact with both Wnt signaling and the NF-kβ pathway.

### 2.4. In Vitro Molecular Efficacy of STW 5

To better understand the protective effect of STW 5 in IBS through a modulation of Wnt signaling, we investigated the specific immune response of EP and ENS cells, using qRT-PCR. In primary ENS cell cultures, DSS-mediated induction of IBS-like conditions promoted an inflammatory state driven by the activated expression of NF-kβ target genes, including *il1β*, *il6*, *il8*, and *tnfα* (Figure 3B). In parallel, we detected a significant reduction in the level of *il10* mRNA. In DSS-induced cells, STW 5 treatment negatively regulated (*p* ≤ 0.01) the expression of the pro-inflammatory cytokine genes *il1β*, *il6*, *il8*, and *tnf*α, and in parallel increased the expression of the anti-inflammatory gene *il10*.

The attenuation of the inflammatory response exerted by STW 5 was less effective in EPc (Figure 3C). After DSS treatment, EPc levels of *il1b*, *il6*, and *il8* mRNAs increased, while their expression of *il10* was significantly reduced. STW 5 treatment induced a defensive effect, reducing the expression of the pro-inflammatory cytokines *il1b* and *il6*.

## 3. Discussion

IBS is a heterogeneous multifactorial disorder driven by environmental, psychosocial, and genetic factors [50]. Here we used in vivo and in vitro zebrafish IBS models to investigate whether STW 5 modulates the expression of Wnt target genes. Considering the important roles of the epithelium, connective tissue compartment, and enteric nervous system in the development of disease susceptibility, zebrafish models have the following advantages: they (i) reduce the cost of research, (ii) enable the investigation of innate immunity due to a delayed maturation of adaptive immunity, (iii) allow easy temporal control over microbial and chemical interventions, and (iv) allow host–microbe interactions to be manipulated for a better understanding of the pathogenesis of gut inflammatory diseases [51]. Chassaing et al. [52] and Scanzi et al. [37] have treated mice with 0.5% DSS in drinking water to impair intestinal homeostasis. In our experiments, zebrafish were treated for only 8 h, to avoid severe inflammation. As confirmed by macroscopic examination and qRT-PCR analyses, the animals responded to DSS by developing intestinal redness, swelling, inflammation, and increased *il8*, *tnfa*, and *wnt3a* mRNA levels. In both EPc and ENSc, the expression of the inflammatory gene *il6* was also increased, confirming the activation of the inflammatory system or a diminished ability to suppress it. Furthermore, as already observed in the small intestine and colonic mucosa of constipation-predominant IBS patients [37], up-regulated expression of *cldn1* mRNA was observed in our study, suggesting a structural alteration similar to that observed in the small intestine and colonic mucosa of constipation-predominant IBS [43].

Cytokine imbalance is a biological marker of IBS [53,54,55]. High levels of IL-1b, IL-6, IL-8, and TNF-α are detected in peripheral blood mononuclear cells in IBS-C and IBS-D [56,57,58,59]. In particular, IL-6 exerts its activity by (i) stimulating submucosal secretomotor neurons [60,61]; (ii) modulating mucosal ion transport; and (iii) regulating epithelial permeability [62,63]. As reported by Dinan et al. [59], the cholinergic system could be involved in increased release of IL-6 in the colon of IBS patients. The chemokine IL-8, which is elevated in human IBS plasma [56,64], acts as a neuromodulator and affects the function of colonic neurons by increasing intracellular Ca^2+^. Both IL-6 and IL-8 stimulate gut contractions in response to alterations in colonic tight junction proteins [65]. In turn, the altered expression of claudins, such as claudin 1 [66], contributes to the dysfunction of the intestinal epithelial barrier and promotes increased gut permeability in IBS patients. The inhibition of IL-6 and IL-8 signaling has been demonstrated to normalize visceral pain sensitivity in a rat IBS model [65].

Our experiments demonstrate that STW 5 is able to reduce the mRNA levels of pro-inflammatory cytokines *il-1b*, *tnf-*α *il-6*, or *il-8* in zebrafish neuronal and epithelial cells, or explanted gut similar to previous findings from different cell types and models [20,67]. Neutralizing IL-6 and IL-8 attenuates evoked neuronal myenteric responses, altered GI motility, and visceral pain sensitivity in an IBS model [65]. Here, the anti-inflammatory activity of STW 5 was also indicated by the restoration of the DSS-induced activity of goblet cells and the normalization of their macroscopic appearance. Therefore, the clinical efficacy of STW 5 demonstrated in IBS could be mediated, at least in part, by this neutralizing effect on pro-inflammatory cytokines.

In the current study, DSS exposure resulted in the upregulation of *cldn1* gene expression in explanted zebrafish gut, suggesting that a structural alteration occurs with IBS inflammation. These findings support a relevant link between inflammation and CLDN1 expression in IBS. Cheng et al. [43] demonstrated increased CLDN1 in the small intestine and colonic mucosa of patients affected by constipation-predominant IBS. This association suggests that the DSS model proposed in the present study can be effective in simulating some forms of IBS, as the upregulation of *CLDN1* mRNA led to reduced intestinal permeability and resulted in constipation in patients.

Notably, *CLDN1* has been described as a target gene for Wnt3a/β-catenin/TCF signaling [43,68,69]. This modulatory activity could explain STW 5-induced downregulation of *cldn1* observed as a result of the negative regulation of *wnt3a* shown in our study. This observation supports the hypothesis that Wnt signaling could be a novel target for the STW 5 herbal preparation.

The interplay between the intestinal epithelial barrier and neuromuscular compartment gives rise to a dynamic network that preserves GI physiology and gut microenvironment integrity. *IL-10* plays a critical role in preventing inflammatory and autoimmune pathologies by limiting the immune response to pathogens and microbial flora [70]. IL-10 is a target gene of NF-kβ and, in response to an inflammatory insult, is transcriptionally upregulated by nuclear translocation of the p50 transcription factor. Accordingly, restoration exerted by STW 5 on the DSS-induced upregulation of *il10* expression in the explanted gut could indicate a protective activity that balances the host immune response and the epithelial secretory activity. In line with this evidence, our data showed that the number of goblet cells accumulating mucus was reduced by STW 5 in different regions of the gut, as previously reported [71,72]. The differential, cell-specific effect of STW 5 was clearly observed in its ability to promote the expression of *il10* in ENS cells to balance the immune response and regulate neuronal excitability.

Taken together, these data identify novel targets for STW 5 that substantiate and could explain its clinically proven efficacy in treating IBS symptoms.

## 4. Materials and Methods

### 4.1. Herbal Preparation

Lyophilized STW 5 (5.7 g percent dry residue, batch number 430,392) was generously provided by Steigerwald Arzneimittel GmbH (Bayer Consumer Health, Darmstadt, Germany). The quality of this batch complied with the quality prerequisites for STW 5 (Kroll and Cordes, 2006). STW 5 lyophilisate was dissolved in water and used at the final concentration of 0.12 mg/mL, based on our preliminary studies (data not reported) and demonstrated as effective to induce anti-inflammatory effects starting from 16 h of treatment.

### 4.2. Animals

**Figure 4:**
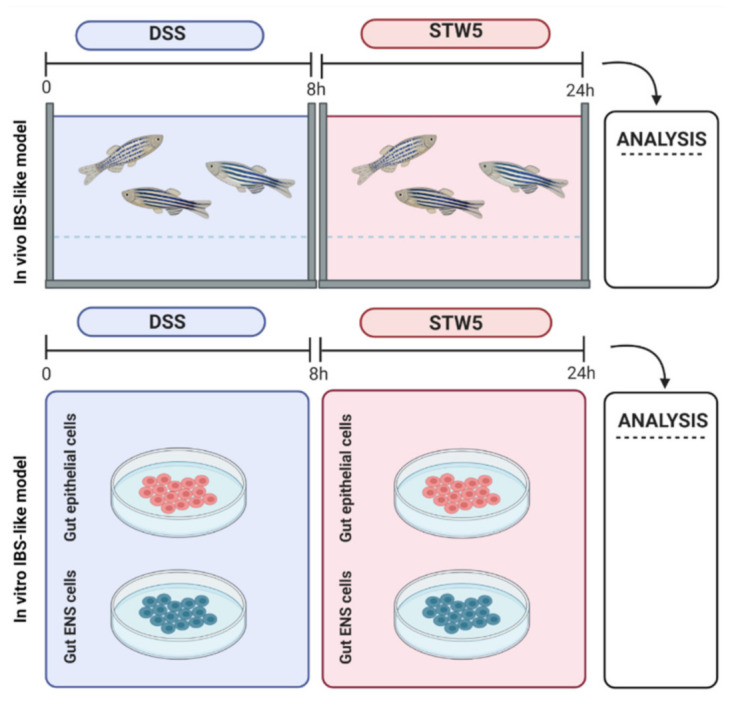
Experimental scheme to obtain in vivo and in vitro models of irritable bowel syndrome (IBS) (created with BioRender.com, accessed on 21 May 2021). (**Top**) In vivo treatment: IBS was induced in zebrafish with 0.5% dextran sodium sulfate (DSS) in the water. The water was exchanged after 8 h, a mixture of nine medicinal herbs (STW 5) was added to a concentration of 0.12 mg/mL, (dissolved in water), and the fish were kept in this for 16 h. The gut was explanted for examination. (**Bottom**) In vitro treatment: The intestine from wild-type zebrafish was dissected and digested to obtain cells from the epithelial compartment (EPc) and myenteric plexus (ENSc). After induction of IBS-like inflammation with 0.5% DSS for 8 h, EPc and ENSc were treated for 16 h with 0.12 mg/mL ethanol-free, lyophilized STW 5 resuspended in FBS-deprived culture medium.

Adult zebrafish (*Danio rerio*, *n* = 41 males, aged 6 months) were housed and fed as described by Aleström et al. [73]. The environment was maintained at 28.5 °C with a 12 h light:dark cycle. The study design is illustrated in Figure 4.

### 4.3. Investigation of Canonical Wnt Components in Zebrafish Gut

To demonstrate that STW 5 suppresses inflammation by modulating β-catenin signaling, a preliminary analysis using immunofluorescence was performed on full-thickness gut wall preparations to detect the expression patterns of Wnt components (i.e., Fzd9 and Wnt3a) in neuronal and non-neuronal compartments. Intestine explants were fixed overnight in BD Cytofix Fixation Buffer (Becton Dickinson Biosciences, San Jose, CA, USA), and embedded in paraffin wax (Carlo Erba, Milan, Italy). Sections of 5-μm thickness were prepared using a Histoslide 2000 microtome (Leica Microsystems, Wetzlar, Germany). After deparaffinization in xylene and rehydration, the samples were immersed in citrate buffer (pH 6) and treated four times (5 min/each) with microwave irradiation. Next, the sections were blocked in 10% bovine serum albumin (BSA) for 2 h at room temperature (RT). To detect target proteins, the samples were incubated with primary polyclonal antibodies against PAN neuronal marker (Merck Millipore, Billerica, MA, USA), Fzd9, and Wnt3a (both from Immunological Sciences, Rome, Italy), followed by staining with Alexa Fluor 488-conjugated or Alexa Fluor 594-conjugated secondary antibodies (Thermo Fisher Scientific, Darmstadt, Germany) (Table 1). For intracellular markers, membrane permeabilization was performed with 0.2% Triton X-100 for 30 min before the incubation with antibodies. Secondary antibody-matched negative controls were prepared as reference. Data were acquired using a DM2000 microscope (Leica Microsystems, Wetzlar, Germany).

### 4.4. Assessment of Zebrafish Models of IBS

#### 4.4.1. In Vivo Model

The IBS-like model was induced by placing adult male zebrafish into individual tanks containing 0.5% dextran sodium sulfate (Sigma-Aldrich, St. Louis, MO, USA) for 8 h [37,74]. After a water change, the animals were treated with 0.12 mg/mL STW 5 for 16 h, and then anesthetized with tricaine (0.16 mg/mL, E10521, Sigma) and sacrificed by decapitation for gut explant. The establishment of a functional in vivo IBS model was evaluated through macroscopic examination of the intestine, histochemistry, and expression analysis of genes associated with general inflammation (*wnt3a*), IBS (*il1β*, *il6*, *il8*, *tnfα*) [75], intestinal permeability (*cldn1*) [76], and immune regulation (*il10*) [77]. Animals kept under resting conditions were used as controls.

##### Histochemistry

To evaluate mucous accumulation, 5-µm thick slices from the bulb, midgut, and hindgut of untreated (control), DSS- or DSS+STW 5-treated animals were incubated with 1% Alcian blue (Sigma-Aldrich, St. Louis, MO, USA) in 3% acetic acid (pH 2.5; Sigma-Aldrich, St. Louis, MO, USA) for 30 min. Nuclear counterstaining was performed by treating with 0.1% nuclear fast red (Sigma-Aldrich, St. Louis, MO, USA) for 5 min. After dehydration in ethanol, the sections were cleared with xylene and mounted with Pertex mounting medium (Leica Microsystems, Wetzlar, Germany). As an increased number of mucus-producing cells is commonly considered to indicate active inflammation, we counted the goblet cells in bulb, midgut, and hindgut explants. The quantification was performed using five different slides of each part of the intestine from three animals (*n* = 15 sections total for bulb, midgut, and hindgut). Data were expressed as the mean number of goblet cells ± standard deviation (SD). Statistical significance was calculated using the Wilcoxon test, comparing DSS-treated or DSS+STW 5-treated samples with controls.

##### qRT-PCR

When STW 5 treatment was completed, intestines were collected in BeadBug prefilled tubes (Sigma-Aldrich, St. Louis, MO, USA) containing 1 mL of TRI Reagent solution (Zymo Research, Irvine, CA, USA). Tissues were mechanically disrupted, and total RNA was extracted according to the manufacturer’s instructions. The amount of total RNA was evaluated by measuring the absorbance at 260 nm with a Nanodrop 2000 (Thermo Fisher Scientific, Waltham, MA, USA). For mRNA purification, 3 µg of each total RNA sample was purified using a Dynabeads mRNA DIRECT kit (Thermo Fisher Scientific, Waltham, MA USA). Quantitative RT-PCR was performed in a one-step procedure using a Magnetic Induction Cycler (MIC) PCR machine (Bio Molecular Systems, Australia), qPCR SyGreen 1-step Go Lo Rox (PCR Biosystems Ltd., London, UK), 7.5 ng of mRNA, and the oligonucleotides (Thermo Fisher Scientific, Waltham, MA, USA) listed in Table 2. The housekeeping gene eukaryotic translation elongation factor 1 alpha 1 (*ef1α*) was used as an internal control. Relative gene expression levels (i.e., fold changes normalized to controls) were quantified using the comparative Ct method (2^–ΔΔCt^). Each target gene was evaluated in triplicate, and three independent experiments for each study group were executed. Significance was determined using the Wilcoxon test, comparing the DSS+STW 5 samples to DSS-treated samples or controls.

#### 4.4.2. In Vitro Models

Published protocols for isolating intestinal cells from adult zebrafish are currently lacking. We devised a method to isolate epithelial and myenteric plexus cells from zebrafish gut by adapting a standardized procedure [78]. Briefly, animals were anesthetized by immersion in tricaine solution. After decapitation, the whole gut was extracted under a dissecting microscope. The surrounding mesentery, fat, and feces were removed, and the remaining tissues were washed several times in phosphate-buffered saline containing 2% antibiotic/fungizone solution (Sigma-Aldrich, St. Louis, MO, USA). Afterwards, the tissues were digested in 4 U Dispase type II solution (Roche, Basel, Switzerland) for 40 min at 28 °C. The samples were then differently processed for (i) the isolation of epithelial cells (EPc), (ii) the morphological characterization of the enteric nervous system (ENS) by whole-mount immunofluorescent staining, and (iii) the isolation of ENS-derived cells (ENSc).

##### EP Cell Cultures

The epithelium was collected from samples digested with 4 U Dispase II solution and then incubated in Accumax solution (Sigma-Aldrich, St. Louis, MO, USA) for 30 min at 28 °C. After tissue dissociation with gentle pipetting, the samples were centrifuged at 1200 rpm for 5 min, and the pelleted cells were used to seed glass coverslips coated with 10 μg/cm^2^ collagen IV (Sigma-Aldrich, St. Louis, MO, USA) in Dulbecco’s modified Eagle medium supplemented with high glucose (Lonza, Basel, Switzerland), 10% FBS, 0.25 U/mL insulin (Sigma-Aldrich, St. Louis, MO, USA), 1% L-glutamine (Sigma-Aldrich, St. Louis, MO, USA), 1% antibiotic/fungizone solution, and 10 ng/mL epidermal growth factor. Images of EP cultures were acquired using a DM2000 microscope (Leica Microsystems, Wetzlar, Germany) equipped with a Nikon Digital Sight DS-5Mc camera (Nikon Corporation, Tokyo, Japan).

##### Whole-Mount Immunofluorescent Staining

After digestion with Dispase II (Sigma-Aldrich, St. Louis, MO, USA), the muscle layer of the intestine was fixed with BD Cytofix Fixation Buffer overnight at 4 °C. For immunofluorescent staining, the samples were permeabilized with 0.5% Triton X-100 (Sigma-Aldrich, St. Louis, MO, USA) for 30 min following a blocking step with 10% BSA for 1 h at RT. Samples were incubated with primary antibody against PAN neuronal marker (Merck Millipore, Billerica, MA, USA) and an Alexa Fluor 488–conjugated secondary antibody. After mounting in Fluoro-Gel II solution containing 4′,6-diamidino-2-phenylindole (DAPI) (Fisher Molecular Biology, Trevose, PA), the samples were analyzed using a Leica TCS SP5 confocal microscope (Leica Microsystems, Wetzlar, Germany).

##### ENS Cell Cultures

When collected as described above, the muscle layer was treated with 5 µg/mL collagenase II, 200 μg/mL DNAse (Roche, Basel, Switzerland), and 50 ng/mL trypsin-chymotrypsin inhibitor (Sigma-Aldrich, St. Louis, MO, USA) in Hank’s buffered saline solution for 50 min at 28 °C. The cells and remaining undigested tissues were then centrifuged at 800 rpm for 5 min. The pellet was incubated in AccuMax solution for 20 min at 28 °C. Finally, the samples were cultured in ENS culture medium composed of Neurobasal-A (Gibco, Billings, MT, USA), 2% neuronal stem cell supplement (Gibco, Billings, MT, USA), 1% BSA (Sigma-Aldrich, St. Louis, MO, USA), 5% FBS (Thermo Fisher Scientific, Darmstadt, Germany), 0.1% ß-mercaptoethanol (Thermo Fisher Scientific, Darmstadt, Germany), 1% L-glutamine (Sigma-Aldrich, St. Louis, MO, USA), and 1% antibiotic/fungizone solution. To optimize the in vitro cell growth, culture medium was supplemented with 10 ng/mL epidermal growth factor, 20 ng/mL basic fibroblast growth factor, and 10 ng/mL glial cell line-derived neurotrophic factor (all from ImmunoTools, Friesoythe, Germany). Twenty-four hours after isolation, the medium was replaced with fresh medium without FBS and the cells were cultured for 7 days at 28 °C in a 5% CO_2_ atmosphere at 95% humidity. Optical microscopy images were acquired using a DM2000 microscope (Leica Microsystems, Wetzlar, Germany) equipped with a Nikon Digital Sight DS-5Mc camera (Nikon Corporation, Tokyo, Japan).

##### Morphological and Immunophenotypic Characterization of EPc and ENSc

Primary EPc and ENSc morphology were analyzed using optical microscopy. After fixation in BD Cytofix Fixation Buffer, EPc were characterized by immunofluorescence for the expression of cytokeratins using anti-Pan Cytokeratin antibody (Abcam, Cambridge, UK) and Alexa Fluor 488-conjugated secondary antibody (Thermo Fisher Scientific, Darmstadt, Germany) (Table 1). ENSc were indirectly stained with primary antibodies against Sox2, Sox10, and a phycoerythrin-conjugated secondary antibody (Table 1). Data were acquired using a BD FACSCanto™ II Flow cytometer (Becton Dickinson Biosciences, San Jose, CA, USA) and FACSDiva v6.1.3 software (Becton Dickinson Biosciences, San Jose, CA, USA). In parallel, controls were prepared by omitting the primary antibody. Results were analyzed using FlowJo software and were presented as percent positive ± standard deviation (SD).

##### Impact of STW 5 on EPc and ENSc

Zebrafish gut cultures were treated with 0.5% DSS for 8 h to induce IBS-like inflammation. After DSS removal, some samples were cultured for 16 h with 0.12 mg/mL ethanol-free lyophilized STW 5 resuspended in FBS-deprived culture medium. In parallel, cells treated with DSS for 8 h and then kept in standard culture medium were used as controls. All samples were examined for the expression of inflammatory mediator genes (*il1b*, *il6*, *il8*, *il10*, and *tnfα)* as described earlier under qRT-PCR in Section 4.4.1. The analysis of data from four representative EPc and ENSc populations was performed as described above.

#### 4.4.3. Statistical Analysis

Data were presented as means ± standard deviation (SD). Statistical differences were calculated using the Wilcoxon test, comparing DSS-treated or DSS+STW 5-treated samples with controls. Results were considered significantly different at *p* ≤ 0.05.

## 5. Conclusions

To our knowledge, this study is the first to suggest that the regulation of Wnt signaling could be a novel mechanism by which STW 5 exerts anti-inflammatory and intestinal permeability-restoring effects. This pharmacological activity is postulated to be dependent on the ability of STW 5 to regulate the expression of *cldn1* and *wnt3a* and interfere with Wnt signaling, without affecting their physiological roles in normal tissues.

## Figures and Tables

**Table 1:** Antibodies used in flow cytometry and immunofluorescence studies to investigate the effects of STW 5 in zebrafish IBS. Due to the high genomic similarity of zebrafish with human, with zebrafish sharing 70% of their genes with humans, anti-human antibodies were used to detect fish markers when no specific antibodies were available. AF: Alexa Fluor; FZD9: Frizzled 9, a Wnt-signaling receptor; PAN cytokeratin: epithelial cell marker; PAN neuronal: neurofilament marker; PE: phycoerythrin; Sox2: transcription factor; WNT: signaling protein involved in inflammation.

Primary Antibody	Manufacturing Company
Rabbit anti-FZD9	Immunological Sciences
Rabbit anti-WNT3A	Immunological Sciences
Mouse anti-PAN Neuronal	Merck Millipore
Mouse anti-PAN Cytokeratin	Abcam
Mouse anti-Sox2	Santa Cruz Biotechnology
Rabbit anti-Sox10	Santa Cruz Biotechnology
Secondary antibody	
Goat anti-rabbit AF488	Invitrogen
Goat anti-mouse AF594	Invitrogen
Goat anti-rabbit PE	Santa Cruz Biotechnology
Goat anti-mouse PE	Santa Cruz Biotechnology

**Table 2:** Oligonucleotide primers used for quantitative RT-PCR analysis of gene regulation in response to STW 5 treatment in irritable bowel syndrome. F: forward; R: reverse.

Gene Name		Primer Sequence (5′-3′)	Accession Number
eukaryotic translation elongation factor 1 alpha	*ef1α*	F: TTCGAGAAGGAAGCCGCTG	AY422992
		R: CAGCAACAATCAGCACAGCAC	
wnt family member 3A	*wnt3A*	F: GGCGACTACATGAAGGACAA	AY613787.1
		R: TACTTTGGCCGTAGGGTTTC	
claudin 1	*cldn1*	F: CGCCACAGGTGAAGAGTAAA	NM_131770.1
		R: CCTCGCGTTAGTTGGAGTAAA	
tumor necrosis factor α	*tnfα*	F: CCGTCTGCTTCACGCTCC	AY427649.1
		R: GTCTTTGATTCAGAGTTGTATCC	
interleukin 8	*il8*	F: TGTTTTCCTGGCATTTCTGACC	XM_009306855
		R: TTTACAGTGTGGGCTTGGAGGG	
interleukin 6	*il6*	F: GCTACACTGGCTACACTCTTC	NM_001261449.1
		R: GAGACTCTTTACGTCCACATCC	
interleukin 10	*il10*	F: CTCTGCTCACGCTTCTTCTT	NM_001020785.2
		R: GCTCCCTCAGTCTTAAAGGAAA	
interleukin 1β	*il1β*	F: GACATGCTCATGGCGAACG	AY340959.1
		R: GCAAATCGTGCATTGCAAGACG	

## Data Availability

Data is contained within the article.

## Abbreviations

AF: Alexa Fluor; CLDN: Claudin; DSS: Dextran sodium sulfate; ef1α: Eukaryotic translation elongation factor 1 alpha 1; ENS: Enteric nervous system; EPC: Epithelial cells; FZD9: Frizzled 9; IBS: Irritable bowel syndrome; IL: Interleukin; PE: phycoerythrin; Sox2: SRY-Box transcription factor 2; TNF: Tumor necrosis factor; WNT: Wingless-related integration site; 5-HT: Serotonin.
